# Editorial Chit-Chat

**Published:** 1876-10

**Authors:** 


### Editorial Chit-Chat.

THEODORE.—We knew he would tilt-on to something that would upset him at last.

HAIL TO THE KING.—Prince Milan has been proclaimed King of Servia. They Servia noble prince, right in so doing.

LOST VOTES.—We overheard a discussion between two honest voters who declared their intentions not to vote for Tilden, because they believed he and Moulton acted dishonestly in "that Beecher matter."

FOUNDERED.—The papers announce that the steamship Canadian has foundered upon a rock, rendering the vessel a total loss. We have seen it stated somewhere that an edifice founded upon a rock was rendered safer than otherwise.

HUXLEY.—Rev. Thos. K. Beecher has gone to New York to attend this noted scientist's lectures. Mr. Beecher will doubtless review and criticise the lectures for the benefit of his people, which will be quite as well as to hear Huxley himself.

MAJ. TOWNER'S POEM.—The poem delivered by Maj. Towner at the State Editorial Association, has been printed in pamphlet form, and a very neat job of printing it is too. Both the poem and the printing are worthy of their respective authors.

QUEER TREE.—In the "buttonwoods," at the ower portion of the Fifth ward in this city, may be seen a tree, which seems to be a buttonwood at the trunk, but at fifteen feet above the ground has two immense limbs, one a buttonwood and the other an elm. Drive down and look at it—it's queer.

CAMPING OUT.—As is our yearly custom, accompanied by our constant piscatorial friend H., we encamped for three weeks upon the banks of the charming Lycoming. As it rained every blessed day but one during the entire time, our recollection of the affair is not of the most pleasant character. However, we did have some enjoyable moments, when, between the thunder storms, we succeeded in bringing some gamey trout to creel.

For the comfort and peace of mind of our friend, we sincerely hope that the future world may be unclouded with thunder storms; for, from a discussion of the entire range of metrological possibilities, we are convinced that H. had rather remain at home than hazard a vague probability even, of encountering thunder and lightning in the blissful spiritual world.

"SCHOONER."—When you visit the centennial exhibition and feel inclined to relieve your thirst at some lager beer fountain, let not your friends enveigle you into calling for a "schooner." H. declares he would founder, sink, or perhaps become a total wreck, in an endeavor to encircle another.

---

MODEL CITY.—Dr. Richardson, of London, England, has planned to lay out a city, near Sussex, which shall be entirely and absolutely healthful. The system of drainage and water, and the style of house, so far as ventilation, light and shade is concerned, will all be controlled by the doctor.

---

CALIFORNIA JACK.—There resides an otherwise very amiable lady in this city, in whose presence it is not safe to suggest the playing of that game in which the ace captures the jack. She declares it to be too harrassing to one's peace of mind to carry the jack and deuce almost to the end of the game, to have the former captured with the queen and the latter with a tray. Although an opponent may have a high time capturing her low, none but a jack would make game of her for the misfortune.

---

CHARACTER.—Few people have any now, to speak of, because these be political times. We never knew before how many infernal scoundrels were at large in the land,—why, Tweed is an archangel in comparison with them. Herein consists the beauty of American politics—it brings all the villains to the surface. Having read the political papers of both sides, for a month, we are amazed that a vigilance committee has not been organized long ere this, to hang those two conspicuous ruffians and criminals—Hayes and Tilden.

---

HORRIBLE.—Seated upon a rug, from beneath whose blended colors, limbs of graceful outline lay half concealed, while upon her shoulders of snowy whiteness, rested ringlets of her golden hair, and her face—of angelic sweetness, with blue eyes sparkling from beneath their long and graceful lashes, rolicked this maiden—so comely of form and feature that angels must have poised their wings to look upon her beauty. In her arms an infant lay, the maiden toying with it the while, as would a mother with her darling; when suddenly, as though some fiend had her possessed, she placed the infant's toes to her rosy lips and crunched them into nothingness between her pearly teeth! And, more horrible still, before the helpless infant had time to scream or make its direful situation known, or even ere we could our friendly hand outstretch, she dashed its brains out upon the hearth and then sat calmly plucking its silky tresses from the fragments of skull strewn round! And then did she frolic and laugh and clap her dimpled hands, as though an innocent act of mirthfulness had been performed, until her mother—our wife—upon the scene appeared and with hands uplifted, in holy horror, as it were, proclaimed—why, my dear, how came you to let her smash her dolly so? To which we mutely answered not a word, but picked the fragments from the floor and casting them into the grate, hugged the little vixen to our bosom and blest our stars 'twas not *she* that met this dire calamity.

---

SPIRITUAL MANIFESTATIONS.—They seem to be at a discount now—under a cloud as it were, with the confounded cloud laden with thunder-bolts that are knocking cabinets, mediums and spirits into dire confusion, or more poetically, perhaps, into a cocked hat. Those Rochester editors seem to have no mercy, much less respect, for the acrobatic performances in so-called spiritual manifestations, and are just "going for" the aforementioned jugglers. Jennings—an acknowledged powerful medium, who did marvelous things in the manifestation business, stirring up the wildest exclamations of delight among the faithful followers of this weird and ghostly occupation, who, after a year of successful deception of his dupes, both in this city and Rochester, reaping a somewhat profitable reward, considered in the pecuniary light, finally came to grief at the hands of a much too skeptical newspaper man, who ruthlessly caught the wilful spirit-chap at his cute ledgerdemain, and exposed him to the wrathful criticisms of his outraged audience. The only thing left for the medium to do, was to make a clean breast of the affair, which he did, handsomely, in two or three columns of the Rochester papers, fully showing how all his exploits were accomplished by the aid of a confederate in an adjoining closet.

At this denoument, are the spiritualists justly indignant, swearing terrible vengeance upon their former prophet, who has sought exemption from punishment in prompt departure to unknown parts. Following up this exposure, came that also of a Mrs. Markee, formerly of Havana, in this state, where she did a thriving business in cabinet manifestations, materializing the spirits of the friends of any living person who was willing to pay a dollar for such an exhibition. But while she thrived excellently well in the village of Havana, Rochester, with that editor skeptic, who had such a penchant for investigating the deeds of dead men, made it an exceedingly dangerous ground for this bold female medium. Like Jennings, she made a miserable failure of the whole materializing business, that will bankrupt and drive to despair, every other trafficer in this line. While she was exhibiting her choice collection of miscellaneous spirits—duly materialized—to a happy throng of dollar-a-headers, this editor man, who writes for the Rochester *Chronicle*, and who takes no stock in spirits materialized in that form, actually clasped a spirit in his arms, as she emerged from the cabinet, and held her tightly, while she kicked, screamed, fought, scratched like fury, but all to no purpose, for when the lights were turned on, behold, there in the arms of the skeptic, grimaced the medium herself, so exposing another audacious swindle.

It would seem that a few wholesome exposures of this character, would reduce the number of believers in this sort of nonsense—but it doesn't. The same auditors, doubtless, are quite as ready to receive and bless the next juggler humbug that comes their way, and pay their dollars all the same for the extreme satisfaction of being swindled.

Now, we know nothing about "spiritualism" as a religion, and desire not to attack it in that phase of the subject, for we know many excellent persons who seem to have faith in it, and reap much consolation therefrom. We respect their feelings in the manner, but must protest against this manifestation business. It is a cheat and a fraud in every instance. We except no operator in it, neither do we respect him or her. They are swindlers, and they know it, and if we cannot duplicate every trick they perform, and accomplish it through natural and well known laws too, we will agree to forfeit five hundred dollars for our failure. And what is more, we will pay the same sum if we fail to point out the secret means by which so-called manifestations are accomplished, if we are permitted a thorough examination of apparatus used, as well as the apartments in which such ledgermain is performed.

We have no other interest in this matter, than a natural desire to expose fraud and deception wherever we may find it. Trot out your spirits!

---

SPECTACLE VENDERS.—To-day a pink colored circular was thrust under our door, as we suppose it was under everybody's else door in the city, announcing that PROF.—only think of it—Professor H. Jones, Oculist, from Plymouth, England, would call at our house prepared to fit our eyes with his "Brazilian Rock Crystal Spectacles," when he would "adjust our aberation," and cure our "cataracts, films, &c., with his world renowned spectacles." Now, this is an opportunity not to be heedlessly thrown away. When a real, live professor, who has "paid special attention to myopia or near-sightedness, amaurosis or gradual decay of sight," comes to one's door, why, you see, it is something unusual, not to say remarkable. It is not often we are so favored by PROFESSORS—they are such a sensitive, retiring set of fellows, and so studious withal—supposed to be concealed behind a pile of books in their studies, delving into the mysteries of science, or seeking to develop some newly discovered fact not hitherto known to us outside mortals, that when they *do* come out of their holes—so to speak, and with a pack of spectacles upon their backs, at that—why, confound it all, it's exasperatingly funny.

We squandered an entire hour waiting for that Professor, and when, a dark-skinned, black-haired, crooked nosed, humped back, little chap put in an appearance, and tried to pass himself off for the Professor, of course, we readily detected the fraud, and resolutely told him of our suspicions in the matter.

Would you believe it?—that vile personator of intellectual endowment absolutely became offended—or perhaps mad would more aptly represent his true condition—when we casually remarked, with just a shade of malice, perhaps, lingering somewhere, concealed beneath our moustache, that he was an abominable swindle, seeking a price for very indifferent quality of spectacles, four times in excess of what our own spectacle dealers would charge for a better article. But he's gone, and may joy attend him in his migrations in search of ninnies, silly enough to purchase spectacles from a traveling "professor."

---

CENTENNIAL EXPOSITION.—To issue a journal at this time without referring to the Centennial, would be a breach of confidence for which the community would not readily excuse one. Of course, we have been to the exposition, and we hope that all our readers who are able to do so, have gone likewise. It is a wonderful exhibit—the largest fifty-cent show on earth—one that well repays us for the time, money and fatigue incident to its visitation. Perhaps nowhere is a better opportunity afforded to study the oddities of human nature than at this exhibition. So vast is the display of all manners of things, that the tastes of all classes and conditions of men are fully gratified, while the study of the people who congregate there, affords as much amusement and entertainment to the observing man, as does the great exhibition itself. Different people see the same things through separate eyes, and the aggregate of what they *all* see, as related individually, makes a multiplicity of exhibitions truly marvelous to the careful listener as well as observer.

As for ourself, we saw with both eyes wide open, and can briefly relate what made a lasting impression upon our minds. We saw—indeed we could not well avoid it—the "American Soldier" in front of the main entrance, twenty-one and one-half feet high. He was wrought in granite and was a well executed piece of work. Passing him by, we entered the main building, and encountered a bewildering display—one that set our capabilities at naught, and at once determined us to only run through the building, note that which we cared to examine most, and then make several subsequent visitations for careful inspection. But, while upon our run, we found ourself lingering here and there to look upon articles that challenged our admiration—such as Franz Heiss' mearshaum pipes, beautiful specimens of the most dangerous of companions,—the marvelously wrought silver vases of the Gorham Company, particularly the "Century Vase," manufactured from 2,000 ounces of solid silver, containing upon its surface numerous groups of fruits, flowers, animals and human figures, so artistically arranged and beautifully modeled, as to engage hours of your attention in its admiration. Passing on, we soon encountered J. E. Caldwell & Co.'s magnificent cabinet of gems, in which was shown a necklace of diamonds valued at $25,000, and by its side, two sparkling solitaire earrings for $7,000. Almost equal in beauty were the opals of Tiffany & Co., a more delicate, modest and beautiful gem could not grace the neck of a lovely woman. Near them stood the beautiful prize cups of the Yacht Clubs—marvels in workmanship and artistic skill. Glass and porcelain ware abounded everywhere, the coloring and cutting of which manifested the perfection of man's skill in administering to the wants of our æsthetic nature. Fabrics of silk and wool, beautiful of color and pleasing of design, greet your eyes at every turn. Laces—*such* laces, that only the poverty of your exchequer forbids your seizing upon for ornamentation of wife or daughter. Bronzes, from Japan, Germany, France, England and America, vied with each other in claiming your admiration. Furniture and other household comforts, in rich profusion, from all parts of the world, offered you an opportunity for furnishing a home that a prince would envy. Strange are many of the fabrics and utensils exhibited by distant nations, giving one such an insight into their every-day life as could otherwise only be obtained by extensive travel. Weeks could be profitably spent in inquiring into the details of the manufacture of the many interesting and queer objects seen upon every hand. Becoming weary of foot as well as of eye, you depart from the building and take a seat in the neat little cars that carry you about the grounds. In this manner you are able to take in the immensity of the grand exhibition, can see the many beautiful state buildings, and at once award the prize to the state of Michigan for its neat and beautiful cottage—by far the handsomest upon the grounds. Stopping in front of the "Department of Public Comfort," we passed through to the rear of the building, and visited the curious edifice erected by the Japanese, for the purposes of a bazaar. The building is characteristic of the Japanese and contains numerous articles of their own manufacture, which are offered for sale, mostly at very exhorbitant prices. At one side of the bazaar is a Japanese garden, surrounded with a native fence and containing plants and flowers peculiar to their country. Lounging about the place are seen Japs themselves, clothed in American habiliments, having sacrificed their pigtails in order to wear the high silk hat. Much out of place do they look in such raiment, and with their sprouting moustaches and side whiskers. Having strolled among the flowers in front of Horticultural Hall, we refreshed ourself with a cup of delicious coffee, obtained at the Vienna Bakery. Then we inspected Machinery Hall, and among the thousands of curious and wonderful machines there seen, we dwelt longest by "Lockwood's Automatic Envelope Machine," which cuts, folds, gums, counts and packs 120 envelopes in one minute. All that seemed to be lacking in this ingenious contrivance was its inability to talk and give the price of the envelopes it was so rapidly supplying. But for the most intricate, complicated, puzzling and wonderful machine, commend us to the silk weaving loom, exhibited by Thos. Stephens, of Coventry, London. This wonderful loom produced elaborately lettered and illustrated ribbons, having upon them views of the exhibition buildings, that would rival the skill of an accomplished artist, yet, all done by an inanimate *machine*. It seemed incredible. Tack, pin, screw, nail, type and pen making machines were turning out their products, while all other conceivable machinery was contributing their everlasting din to the general confusion. From the mammoth Corliss engine, which ran with a smoothness and gentleness that concealed its immense power, to the little, noisy percussion engine, were various motive machines wonderful of construction—ingenious in design. This department alone, as an educator of our youth, is invaluable. The capabilities of man, and of our nation, as here exhibited, is an achievement of which we may well be proud, giving us an inkling of what may yet be accomplished in the century to come.

We took two days for the art gallery, and stood amazed at its very threshold, for there, on either side of the entrance, stood two immense winged horses, the clumsiest, most awkward, and badly modeled pieces of statuary that ever disgraced an art gallery. They are simply monstrosities, of the heroic type, without the merit of being artistic. Inside, we found many fine paintings, a larger number of very bad ones, and a very small number of meritorious statuary. A few we noted as being worthy of study. First, in the U. S. Department—What the Sea Says, No. 72. Battle of Gettysburg, 168. We recommend this painting particularly, as exhibiting what few battle scenes ever do—the real conditions of affairs upon the battle field. No orderly line of battle, with soldiers in straight rows, as though upon dress parade, but a confusion of men, horses and artillery that exhibits a scene that every soldier will recognize. The Mountain of the Holy Cross, No. 196. Portrait, No. 202. The Stampede, No. 406. The Anatomist, 792. The Annunciation, 794. Yankee Doodle, 866. Four fish paintings, Nos. 897-8-9 and 900, which, for faithfulness of color and portraiture of fishes, surpass anything we have ever witnessed. Norwegian Waterfall, 1024. Genevieve de Brabant, 1068. Two panels of grapes, 1070, the very best still life pictures in the exhibition. Study them and remember that the panels themselves are not real, but are the result of the artist's skill. Chariot Race, 1289 a magnificent production; witness the action of the horses—their different expressions, their muscular developement and superb coloring; a sublime picture that will bear an hour's scrutiny every day. In the department of Great Britain, stop first in front of the greatest picture, the marriage of H. R. H., the Prince of Wales, No. 47. The faces are said all to be portraits, and the painting shows a carefulness in all the details of the picture only attainable by a great artist. The Railway Station, No. 48. Banquet Scene from Macbeth, No. 117; observe the fine grouping, the various expressions of countenance, and the spilled wine upon the marble floor. The Three Jolly Post Boys, 113. Sampson and Delilah, 150, remarkable only for its showing of muscle. In the French Department, Empress Josephine and Family, No. 14. Views in the Tyrol, 43. Death of Julius Cæsar, 63; Rizpah, 76—a picture possessing many pleasing points, the central figures of woman and bird exhibiting fine drawing, good coloring, and strong action. The Bather, 268, a fair, nude picture, not remarkable, but perhaps the best in the collection. The exhibition is singularly deficient in studies of the human form, a fact that does not speak well for our artistic taste. Cours Venion, 289. The First Mourners, 320. German Department, Capitulation of Sedan, 134 and that is all. Our German friends did not favor us with any of their fine paintings. We cannot help but think they could do better than their exhibition would indicate. Austria has nothing worthy of note. Belgium, one picture, No. 58, entitled "Using the Life Boat." Netherlands, Denmark, Sweden, Norway, Italy, Brazil, Argentine Republic, Mexico, Spain and Russia, absolutely nothing worth mentioning, either in painting or sculpture, save Italy in sculpture, whose notable works are as follows: Brotherly Love, No. 1. A Child's Misfortune, 28. The Discarded, 243. After the Bath, 251. Last Days of Pompeii, 329. The Forced Prayer, 332. Finding of Moses, 344. In gallery B., U. S. No. 1190 Thetis. We were disappointed in the exhibit of sculpture. We never saw so poor a collection in any gallery of any pretension. Why Italy did not send some of her best works for our inspection, cannot be answered, unless she thought us incapable of appreciating them. The numbers we have given above correspond with those found in the Official Catalogue, sold in the gallery. Perhaps, by spending more time among the pictures, we could have found more to please us, but the above were all we selected after a two days' search. What has pleased us may not our friends, and doubtless many will differ with us in judgment, but notwithstanding, you will find it interesting to search for these, as you pass along, that have been admired by some friend who has been there before you, and we do not expect you to agree with us that most of the paintings are very, very, poor. We hope you will not, but go prepared to enjoy all you see.

And now, having written already beyond the limits that should be allowed the subject, we drop the whole matter until some future time. While you are reading this, we will be spending another week at the exhibition, making our observations more critically and carefully, when we expect to see many things that escaped our notice before, and enjoy again the articles and pictures that then charmed us, and if we find you have been interested in what we have written, perhaps we will write of other matters which may attract our attention while there.

